# Global burden, trends, and projections of five major upper gastrointestinal diseases, 1990–2046: an analysis of GBD 2021

**DOI:** 10.3389/fpubh.2026.1898446

**Published:** 2026-10-15

**Authors:** Xiru Liang, Yutong Cheng, Qiuai Shu, Ziwei Wang, Haitao Shi, Ning Xie, Yan Cheng

**Affiliations:** 1 Department of Gastroenterology, The Second Affiliated Hospital of Xi’an Jiaotong University, Xi’an, Shaanxi, China; 2 Key Laboratory of Gastrointestinal Motility Disorders of Shaanxi Province (Digestive Disease Laboratory, The Second Affiliated Hospital of Xi’an Jiaotong University), Xi’an Jiaotong University, Xi’an, Shaanxi, China; 3 Bioinspired Engineering and Biomechanics Center (BEBC), Xi’an Jiaotong University, Xi’an, Shaanxi, China

**Keywords:** disability-adjusted life years, future projection, global burden of disease, risk factors, upper gastrointestinal diseases

## Abstract

**Background:**

Upper gastrointestinal (GI) diseases, including gastroesophageal reflux disease (GERD), peptic ulcer disease (PUD), gastritis and duodenitis (GD), stomach cancer, and esophageal cancer, pose significant global health and economic challenges. This study analyzes their trends, regional disparities, risk factors, and future projections from 1990 to 2046.

**Methods:**

Using the 2021 Global Burden of Disease database, we analyzed incidence, prevalence, mortality, and disability-adjusted life years (DALYs) across 204 countries, 21 regions, and five Socio-demographic Index (SDI) levels. Temporal trends were assessed with estimated annual percentage change and joinpoint regression. Decomposition analysis evaluated the contributions of population growth, aging, and epidemiological changes to changes in absolute disease burden. Risk-attributable fractions identified socioeconomic and behavioral factors, and Bayesian models projected future burdens.

**Results:**

From 1990 to 2021, global burdens of PUD, GD, stomach cancer, and esophageal cancer decreased, while GERD increased, particularly in middle SDI regions. Regional and sex disparities persisted, with higher burdens in lower SDI areas. Aging drove absolute disease burden in high-middle SDI regions, while population growth dominated in lower SDI regions. Smoking and alcohol significantly contributed to upper GI cancers. Projections suggest continued GERD increases and a modest PUD rebound, especially in aging and resource-limited populations.

**Conclusion:**

Evolving upper GI disease patterns highlight persistent health inequities. Targeted public health interventions are essential to mitigate future burdens and promote equitable digestive health outcomes globally.

## 1 Introduction

Upper gastrointestinal (GI) diseases—including gastroesophageal reflux disease (GERD), peptic ulcer disease (PUD), gastritis and duodenitis (GD), stomach cancer, and esophageal cancer—have imposed significant health and economic burdens (1–4). Non-malignant conditions like GERD and PUD reduce quality of life through chronic symptoms and increase healthcare expenditures and productivity losses (5); while stomach and esophageal cancers are leading causes of cancer-related death globally, especially in East Asia and low-income areas, with substantial economic burdens arising from treatment costs and premature mortality (6–8). The burden of these diseases varies by sex, age, and geography. Men have higher rates of upper GI malignancies and PUD, while GERD prevalence has increased among younger adults and rapidly developing regions. From 1990 to 2021, global age-standardized rates (ASRs) of PUD and upper GI cancers declined with advances in prevention and treatment (9). Key interventions include *Helicobacter pylori* (Hp) eradication, proton pump inhibitor (PPI) therapy, and endoscopic screening, although access to and implementation of these interventions remain uneven across socioeconomic settings because of differences in healthcare resources and endoscopic capacity (10). GERD prevalence has steadily increased due to rising obesity, dietary changes, and lifestyle shifts (11). Key modifiable risk factors include Hp infection, smoking, alcohol, high body mass index (BMI), and diets high in salt-preserved or smoked foods and low in fruits and vegetables (12–15). These changing risks require updated surveillance and targeted prevention.

Given their substantial health and socioeconomic burden and changing epidemiological patterns, robust and up-to-date data are essential for health policy and resource allocation. The Global Burden of Disease (GBD) 2021 study offers standardized prevalence, incidence, mortality, and disability adjusted life year (DALY) estimates for major diseases in 204 countries, enabling consistent global and temporal comparisons (16, 17). Previous GBD studies have largely focused on individual diseases or narrower disease groups, while integrated comparisons of benign and malignant upper GI diseases remain limited. Moreover, long-term projections and comprehensive analyses integrating decomposition, age-period-cohort analysis, and inequality assessment across these diseases remain scarce (9, 11). Building on previous disease-specific studies, the present analysis uses the updated GBD 2021 estimates and applies a consistent analytical framework to compare major benign and malignant upper GI diseases across the same populations, time period, and epidemiological indicators. This study aims to characterize the temporal evolution and geographic distribution of the burden of GERD, PUD, GD, stomach cancer, and esophageal cancer from 1990 to 2021 at global, regional, and national levels. We further assess disparities by sex, age, and socio-demographic index (SDI), quantify demographic and epidemiological drivers, evaluate major attributable risk factors, and project disease burden through 2046 to inform targeted public health strategies and clinical resource allocation. These five diseases were selected to represent major non-malignant and malignant upper GI conditions modeled as distinct causes in GBD 2021, allowing an integrated comparison across common upper GI disorders and cancers.

## 2 Materials and methods

### 2.1 Data sources and study design

This study used data from the 2021 GBD database to analyze the global disease burden of GERD, PUD, GD, stomach cancer, and esophageal cancer from 1990 to 2021. Data were extracted using the GBD Results Tool, which provides standardized estimates of prevalence, incidence, DALYs, and mortality at global, regional, and national levels (14). Data were extracted on November 14, 2024, using the following parameters: measures of prevalence, incidence, DALYs, and deaths; metrics of number and rate; causes of GERD, PUD, GD, stomach cancer, and esophageal cancer; age-standardized estimates and 5-year age groups from <5 years to ≥95 years; male, female, and both sexes; years from 1990 to 2021; and global, five SDI-level, 21 GBD regional, and 204 country and territory locations. DALY is an indicator that measures the health impact of diseases and injuries, considering both years of life lost to premature death and years of healthy life lost due to disability or illness. All GBD-derived estimates were reported with 95% uncertainty intervals (UIs), corresponding to the 2.5th and 97.5th percentiles of the uncertainty distribution.

The GBD database combines various data sources, including population censuses, hospital records, death registries, disease surveillance systems, and systematic literature reviews. These data are processed using advanced modeling techniques, such as spatiotemporal Gaussian process regression and ensemble modeling, to generate comprehensive and reliable estimates (5). This study fully adheres to the Guidelines for Accurate and Transparent Health Estimates Reporting (GATHER) to ensure the reproducibility of results and methodological rigor (15).

Disease definitions followed the GBD 2021 cause hierarchy and corresponding ICD classifications. GERD, PUD, GD, stomach cancer, and esophageal cancer corresponded to GBD cause IDs 536, 527, 528, 414, and 411, respectively. Detailed case definitions and ICD-10 codes are provided in Supplementary Table S1.

### 2.2 Study variables and scope of analysis

This study examined key variables, including prevalence, incidence, DALYs, and mortality (excluding GERD from the mortality analysis). ASRs per 100,000 population were used to describe disease burden distribution and temporal trends, while age-specific rates were used for age-stratified and decomposition analyses. DALYs were used as the primary measure of overall disease burden, reflecting the combined impact of premature mortality and disability. Absolute numbers were reported where specifically indicated, whereas crude rates were not used in the primary comparative analyses.

The analysis covered global estimates, five SDI levels (low, low-middle, middle, high-middle, and high), 21 regions, and 204 countries and territories. Stratified analyses by sex (male and female) and age group (5-year intervals, from <5 years to ≥95 years) were conducted to explore disease burden heterogeneity. The study period spans 1990 to 2021.

### 2.3 Statistical analysis

As shown in Figure 1, we conducted a comprehensive descriptive analysis of four key indicators—age-standardized prevalence rate (ASPR), incidence rate (ASIR), DALY rate (ASDALY), and mortality rate (ASMR)—across spatial, temporal, and demographic dimensions. Comparisons between 1990 and 2021 evaluated burden by region, sex, and age group, while annual trends were visualized through time-series analyses.

**Figure 1:**
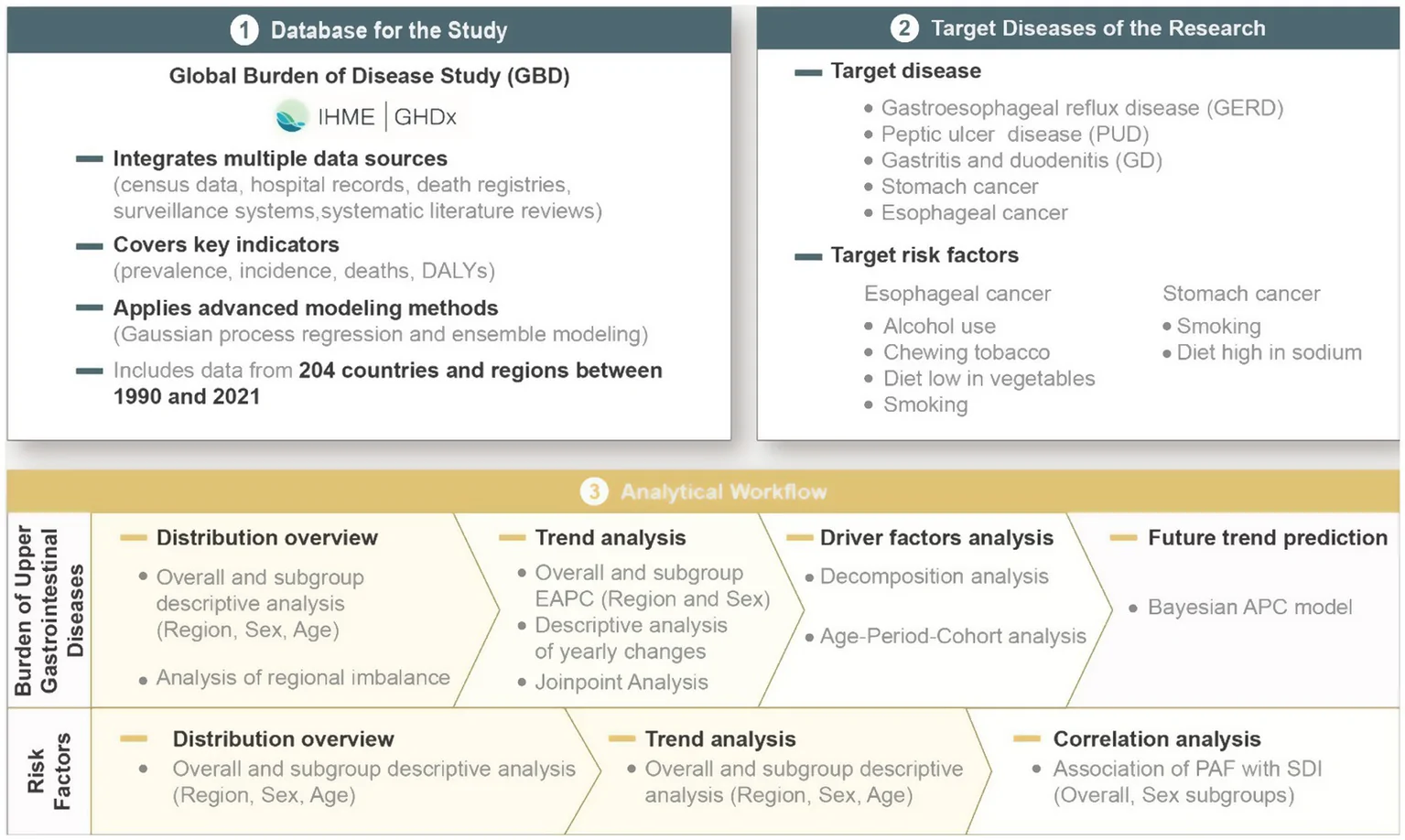
Flow diagram of the research process. SDI, Socio-demographic Index; DALYs, Disability-Adjusted Life Years; PAF, age-standardized population attributable fraction.

To quantify temporal trends in ASRs, the estimated annual percentage change (EAPC) was calculated using a log-linear regression model (18):


ln(ASR)=α+βX+ε

X=calendar year

EAPC=[exp(β)−1]×100%

Joinpoint regression was applied to identify significant inflection points in burden trends. Annual ASRs from 1990 to 2021 were fitted using log-linear segmented regression models. Candidate joinpoints were identified using a grid-search procedure, and the optimal number of joinpoints was selected using Monte Carlo permutation testing, with the number of joinpoints restricted from 0 to 5. For each segment, the annual percentage change (APC) was calculated as (19):


APC=[exp(β)−1]×100%

where *β* represents the segment-specific regression coefficient. The average annual percentage change (AAPC) over the entire study period was estimated by weighting segment-specific trends according to segment length.

Age-period-cohort analysis was further performed using age-specific disease rates from 1990 to 2021 to evaluate the independent effects of age, calendar period, and birth cohort (20). Age was grouped into 5-year intervals and calendar period into 10-year intervals. The median age group and median period group were used as the reference age and reference period, respectively, and period and cohort effects were expressed as rate ratios (RRs) relative to the corresponding reference groups. Age effects describe variation in age-specific rates across age groups at the reference cohort after adjustment for period effects, whereas period effects represent the ratio of age-specific rates in each period relative to the reference period. Cohort effects represent the corresponding rate ratios across birth cohorts relative to the reference birth cohort. Net drift was also estimated to represent the average annual percentage change in disease rates after adjustment for age and cohort effects. Estimates were reported with 95% confidence intervals.

Three-factor decomposition analysis was performed using age-specific rates and population data to quantify the contributions of population growth, aging, and epidemiological change to changes in absolute disease burden (21).

For inequality analyses, countries were ranked from the lowest to the highest SDI, and each country was assigned a population-weighted relative rank based on the midpoint of its cumulative population distribution. The Slope Index of Inequality (SII) was estimated by regressing each age-standardized disease indicator on this relative rank (22):


$$y_{i}=\alpha +\beta R_{i}+\varepsilon_{i}$$

SII=β

where $y_{i}$ denotes the disease indicator and $R_{i}$ the population-weighted relative SDI rank. The regression coefficient *β* represents the SII, corresponding to the absolute difference in the predicted indicator between the highest and lowest ends of the socioeconomic distribution. Population size was used as the regression weight. Heteroscedasticity-robust standard errors were used when appropriate, and SII estimates were reported with 95% CIs. The concentration index(CI) was used to quantify relative socioeconomic inequality and was calculated as (22):


$$\mathrm{CI}=(2/\mu )\sum_{i}w_{i}y_{i}R_{i}-1$$

where $y_{i}$ is the disease indicator, $w_{i}$ is the population share of country *i*, $R_{i}$ is its relative SDI rank, and *μ* is the population-weighted mean. A value of 0 indicates no socioeconomic inequality, whereas negative and positive values indicate a concentration of disease burden toward lower- and higher-SDI populations, respectively.

Finally, we applied a Bayesian Age-Period-Cohort (BAPC) model to project disease burden trends through 2046. The model was fitted to age-specific disease data from 1990 to 2021 and incorporated age, period, and cohort effects within a Bayesian framework. Model estimation was performed using integrated nested Laplace approximation (INLA). GBD population projections were incorporated to estimate future disease counts and rates. Predictions were generated separately by sex, and age-specific rates, crude rates, age-standardized rates, and all-age disease counts were obtained through 2046. Point estimates and corresponding prediction intervals were generated by the model (23).

### 2.4 Risk factor analysis

As shown in Figure 1, we used age-standardized GBD Population Attributable Fractions (PAF) to analyze tobacco, alcohol, and diet risks for stomach and esophageal cancers. PAFs were obtained directly from the GBD 2021 risk-factor estimates rather than recalculated in the present study. Temporal trends (1990–2021), EAPCs, and SDI correlations were evaluated via stratified and regression analyses. Pearson’s correlation coefficient was calculated as (24):


$$r=\frac{\sum (x_{i}-\bar{x})(y_{i}-\bar{y})}{\sqrt{\sum {\left(x_{i}-\bar{x}\right)}^{2}\sum {\left(y_{i}-\bar{y}\right)}^{2}}}$$

Two-sided *p* values were obtained using the corresponding *t* statistic:


$$t=r\sqrt{\left(n-2)/(1-r^{2}\right)}$$

With (n−2) degrees of freedom. A two-sided *p* < 0.05 was considered statistically significant.

All analyses relating SDI to disease indicators or PAFs were conducted at the country level and should therefore be interpreted as ecological associations rather than individual-level or causal relationships.

### 2.5 Data processing and software tools

All GBD-related statistical analyses were performed using easyGBDR (version 2.1.6.1), with additional data processing and visualization conducted in R software (version 4.2.2) using packages such as ‘dplyr’, ‘ggplot2’, and ‘devtools’. The study followed the GATHER guidelines to ensure transparency and reproducibility of data and code.

### 2.6 Ethical statement

This study used publicly available data from the GBD database, which contained no personally identifiable or private information, so ethical approval was not required (25).

## 3 Results

### 3.1 Overview of upper GI disease burden

Upper GI diseases showed marked heterogeneity across disease type, sex, age, and socioeconomic development (Figure 2; Supplementary Figures S1–S3; Supplementary Tables S2–S4).

**Figure 2:**
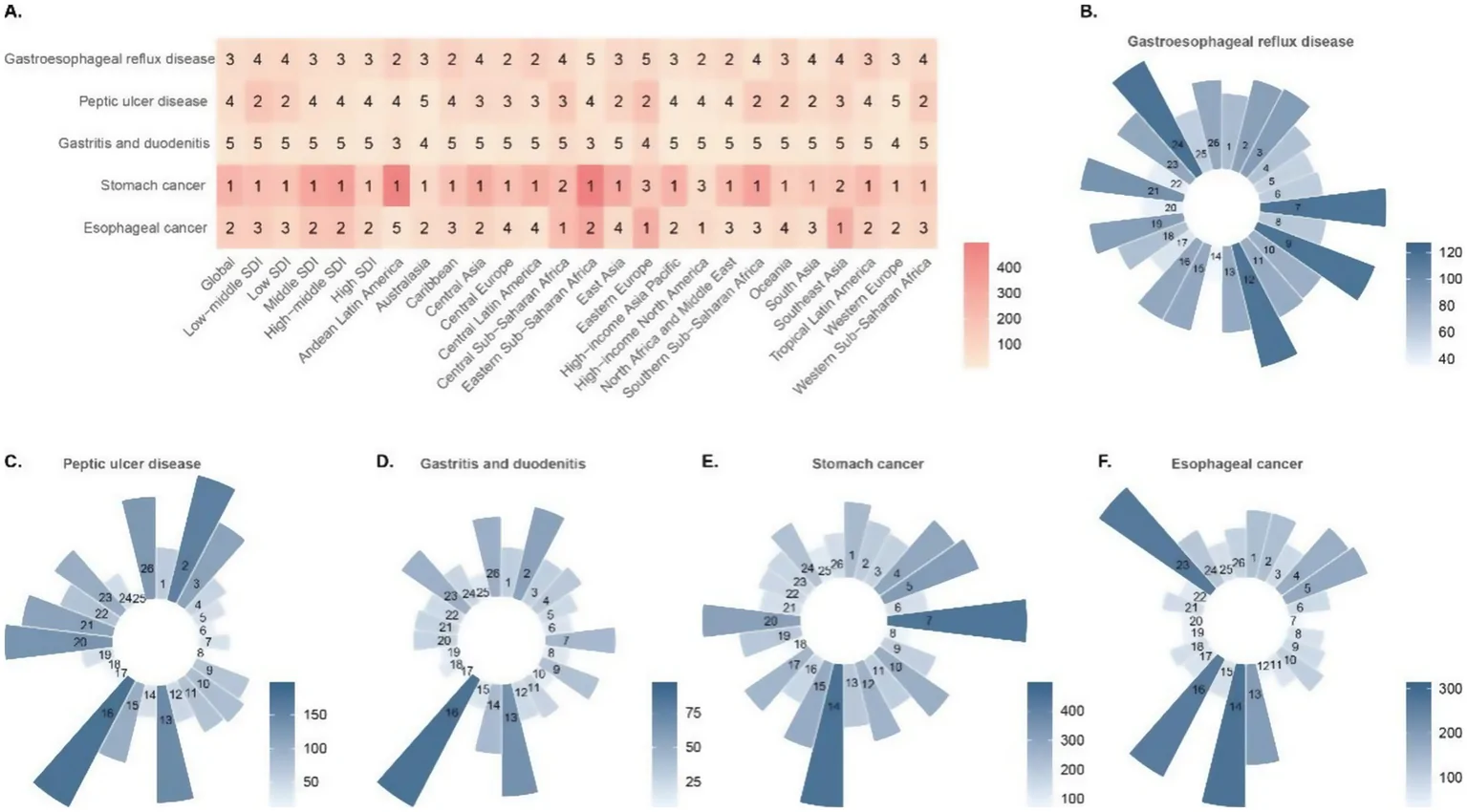
Rankings of the burden of five upper gastrointestinal diseases in 2021 at the global level, across five SDI levels, and 21 regions, along with disease-specific burden rankings by region. SDI, Socio-demographic Index. The disease burden shown in this figure is measured using age-standardized DALY rates (ASDALYs) per 100,000 population. Point estimates are shown in the figure, and the corresponding 95% uncertainty intervals are provided in the Supplementary Tables. Panel **(A)** displays the ranking of disease burdens for five upper gastrointestinal diseases across regions with different SDI levels. Panels **(B–F)** show the disease burden rankings of each individual disease across the various SDI regions.

GERD and GD were more prevalent in females, whereas PUD, stomach cancer, and esophageal cancer consistently showed greater burdens in males. Across diseases, burden generally increased with age, although the age at peak burden differed, occurring earlier for GD and later for PUD and upper GI cancers. Socioeconomic patterns also differed substantially: GERD burden remained concentrated in lower SDI regions; PUD showed contrasting changes in inequality, with disparities in ASMR and ASDALY narrowing but those in ASIR and ASPR widening; stomach cancer showed a shift from higher- to lower-SDI regions; whereas inequalities in GD and esophageal cancer became more pronounced over time. At the country level, the highest-burden locations varied markedly by disease, highlighting substantial geographic heterogeneity (Supplementary Tables S2–S4).

### 3.2 Temporal trends during 1990–2021

Temporal trends differed clearly across the five diseases. GERD was the only condition showing a sustained global increase, with the most rapid rise occurring during 2015–2019 and the strongest increases observed in middle SDI regions. In contrast, PUD, GD, stomach cancer, and esophageal cancer all showed overall declines, with the steepest reductions generally occurring between the mid-2000s and early 2010s. Among these diseases, PUD showed the greatest decline in ASDALY, followed by stomach and esophageal cancers, whereas GD declined more moderately. Sex-specific trends also differed across diseases: the decline in GD was more pronounced in males, improvements in stomach cancer were greater in females, whereas males showed a transient rebound in esophageal cancer burden in the late 1990s. Despite these global improvements, several countries showed divergent trends, indicating substantial regional heterogeneity (Figure 3; Supplementary Figures S4–S6).

**Figure 3:**
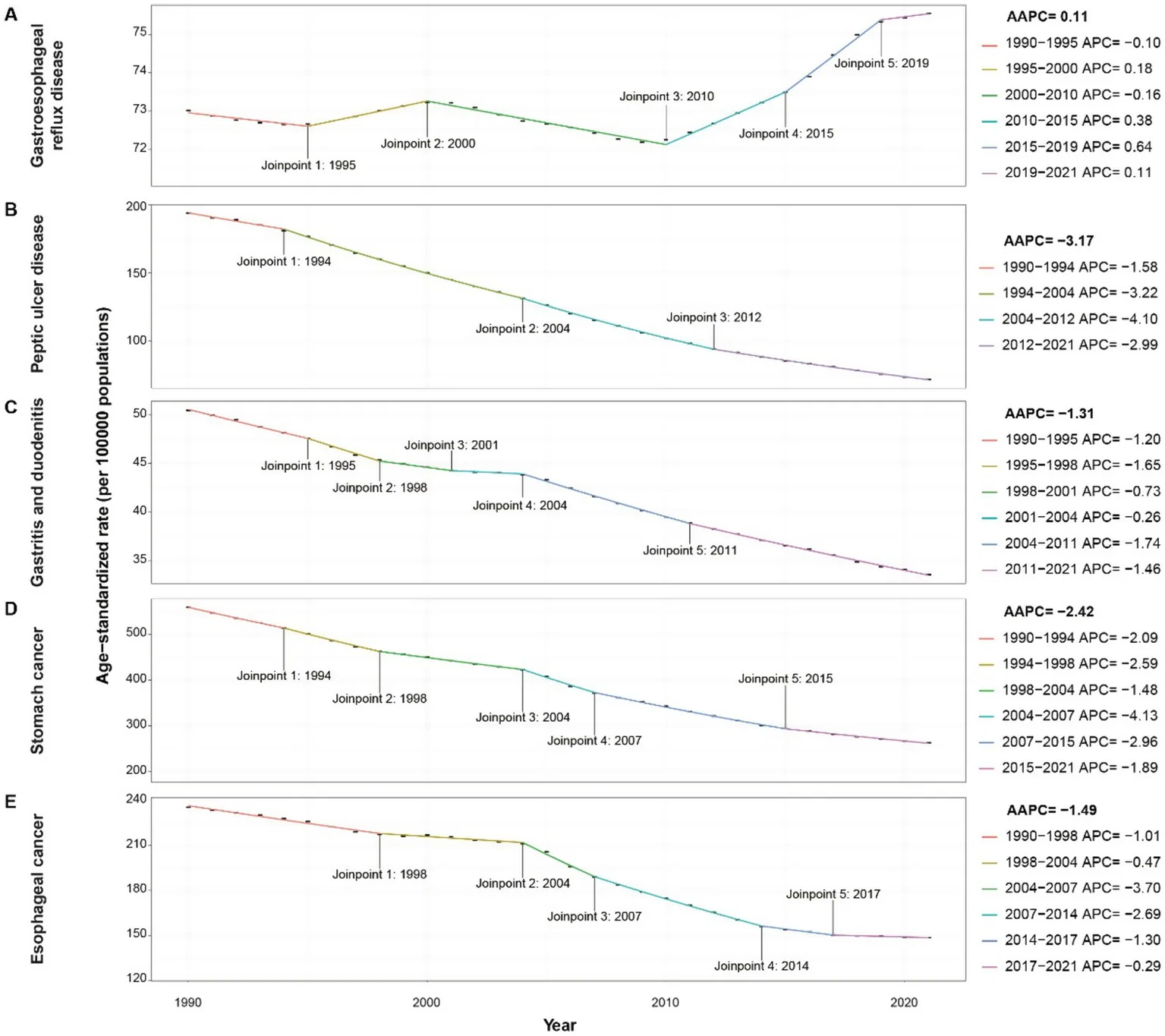
**(A)** gastroesophageal reflux disease, **(B)** peptic ulcer disease, **(C)** gastritis and duodenitis, **(D)** stomach cancer, and **(E)** esophageal cancer. Panels A–E show the corresponding Joinpoint regression analyses from1990 to 2021. ASRs, age-standardized rates; APC, annual percent change; AAPC, average annual percent change.

### 3.3 Drivers of upper GI disease burden

Decomposition analysis revealed divergent patterns across diseases: For GERD, population growth (71.0%) and aging (22.9%) were the main contributors to the increase in absolute disease burden, while epidemiological changes made only a limited contribution (Figure 4A; Supplementary Table S5). For PUD, epidemiological changes contributed substantially to reductions in the absolute number of deaths and total DALYs, whereas demographic changes contributed to increases in the absolute numbers of incident and prevalent cases (Figure 4B; Supplementary Table S5). GD showed a complex pattern: epidemiological changes reduced the absolute disease burden (−210%), whereas the increases attributable to population growth (+200.6%) and aging (+109.5%) outweighed the improvements (Figure 4C; Supplementary Table S5). Reductions in the absolute burden of stomach cancer reductions were mainly attributable to epidemiological changes, especially in middle- and high-middle SDI regions (Figure 4D; Supplementary Table S5). For esophageal cancer, demographic factors, particularly population growth (+192.4%) and aging, were the dominant contributors to changes in absolute disease burden, especially in males (Figure 4E; Supplementary Table S5).

**Figure 4:**
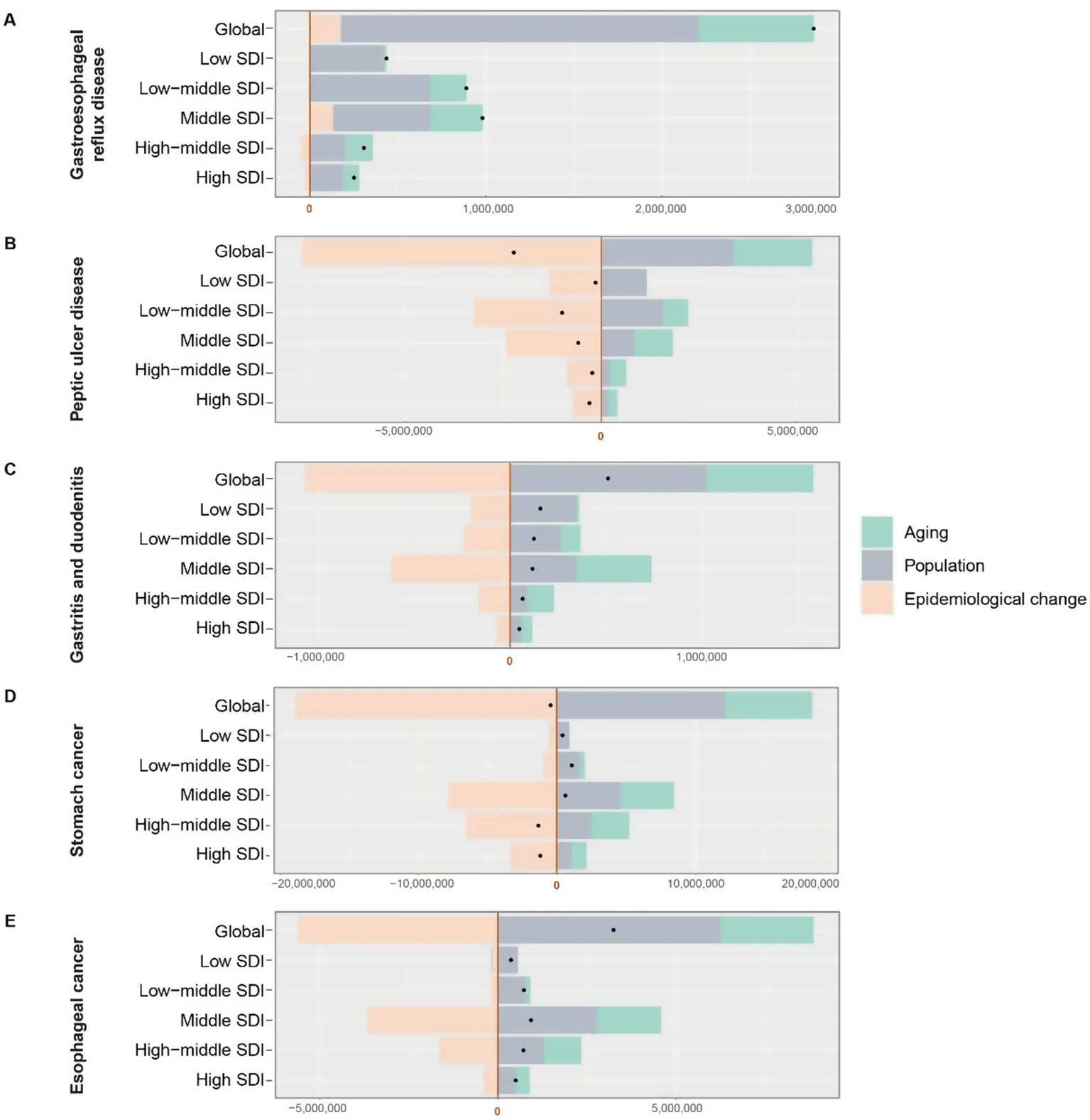
Decomposition of changes in the absolute burden of five upper gastrointestinal diseases at the global level and across five SDI levels. **(A)** Gastroesophageal reflux disease; **(B)** peptic ulcer disease; **(C)** gastritis and duodenitis; **(D)** stomach cancer; and **(E)** esophageal cancer. SDI, Socio-demographic Index.

Age-period-cohort analysis across the five upper gastrointestinal diseases revealed distinct temporal and demographic patterns. GERD differed most clearly from the other diseases, showing a decline in period risk between 2000 and 2010 followed by a rapid increase after 2012, together with a marked rise in cohort risk among those born after 1980. In contrast, PUD, GD, stomach cancer, and esophageal cancer generally showed declining period and cohort effects, although the timing and magnitude of these declines differed. PUD showed a strong age-related increase, with females reaching peak burden later and exhibiting a flatter age pattern than males; its cohort decline became less pronounced among those born after 1980. GD also increased with age, particularly after 60 years, but showed the most evident period improvement during 2012–2021. Compared with the non-malignant diseases, stomach and esophageal cancers demonstrated stronger age and sex gradients, with risk increasing markedly after 50–60 years and remaining consistently higher in males. Stomach cancer showed a clear period decline after 2000 and substantially lower risk in later birth cohorts, although the cohort decline was slower in males. Esophageal cancer showed a distinct period pattern, with relatively higher risk during 1992–1996 followed by a gradual decline, particularly during 2017–2021; cohort risk also decreased progressively across successive birth cohorts, with a more moderate decline in females (Figure 5; Supplementary Table S6).

**Figure 5:**
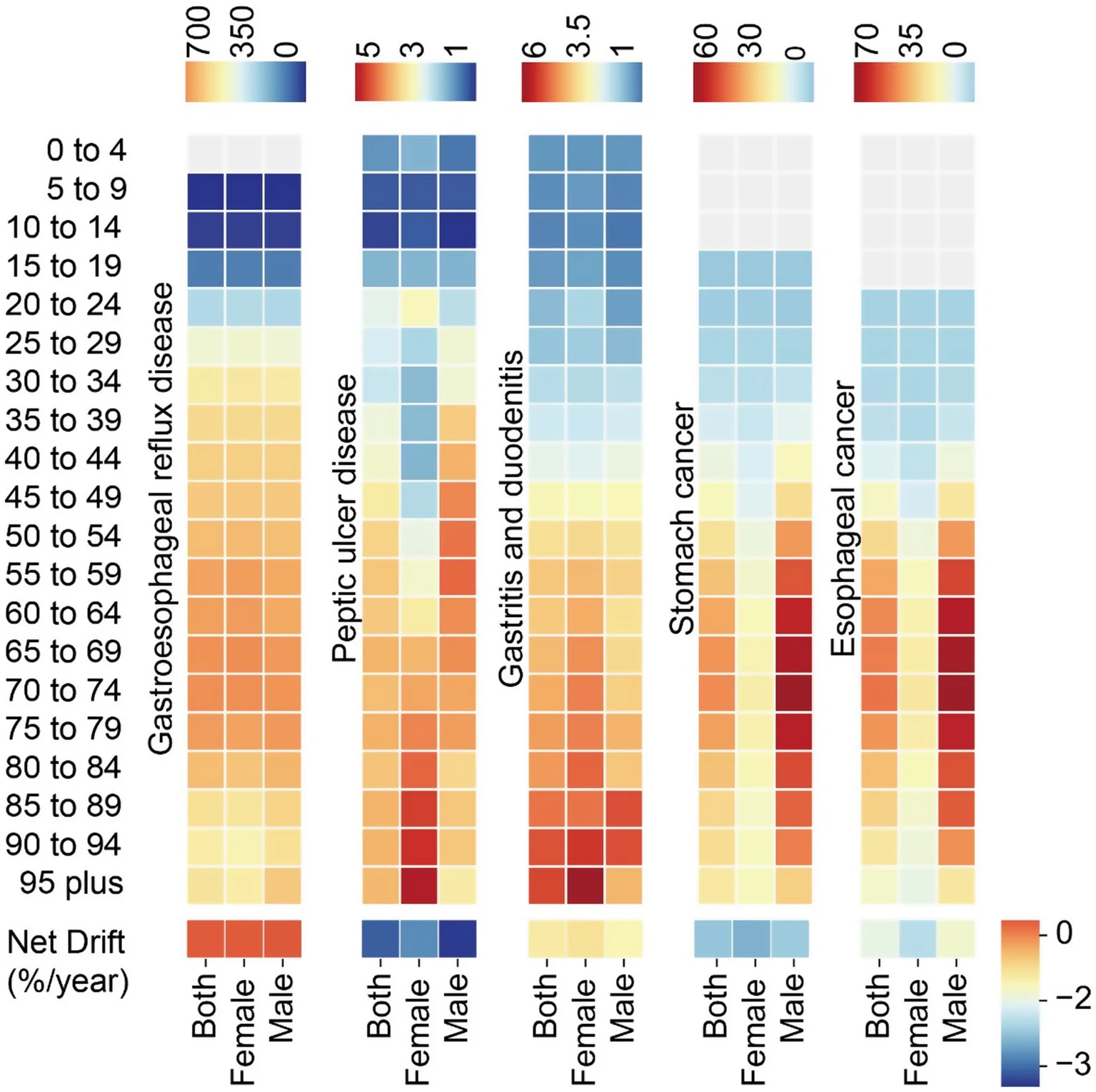
Age-period-cohort analysis of five upper gastrointestinal diseases. Net drift: Overall annual percentage change in disease rate across all age groups, representing the combined period and cohort effects. The age patterns shown in the figure are based on age-specific DALY rates. Corresponding interval estimates are provided in the Supplementary Tables.

### 3.4 Future projections during 2022–2046

Future projections revealed three contrasting patterns. GERD is expected to continue increasing through 2046, with a more rapid rise in males despite the higher overall burden in females. PUD shows a divergent pattern, with continued declines in ASMR and ASDALY but slight increases in ASIR and ASPR. In contrast, GD, stomach cancer, and esophageal cancer are projected to decline across most age-standardized indicators. Sex disparities are expected to persist, particularly for PUD and the two upper GI cancers, with males maintaining the higher burden (Supplementary Figure S7; Supplementary Table S7).

### 3.5 Risk factors and trends

For stomach cancer, smoking and high-sodium diet remained leading global risk factors. Smoking-related PAFs declined from 15.2 to 10.7% (1990–2021), particularly in high SDI regions and males (Figures 6A, 7A; Supplementary Figure S8A). Age-specific burden peaked at 65–69 years. The burden from high sodium diets remained relatively stable across age and sex groups (Supplementary Figure S9A). Esophageal cancer was primarily driven by smoking, alcohol use, chewing tobacco, and low vegetable intake (Figure 6B; Supplementary Figure S8A). Smoking PAFs declined slightly but remained the highest (37.6% in 2021; Figure 7B). Alcohol-related burden increased, especially in high SDI settings, peaking at ages 45–49. The contribution of chewing tobacco rose slightly, particularly among individuals aged 30–44 in South Asia. Notably, dietary risks declined steeply in East Asia (Supplementary Figure S9B).

**Figure 6:**
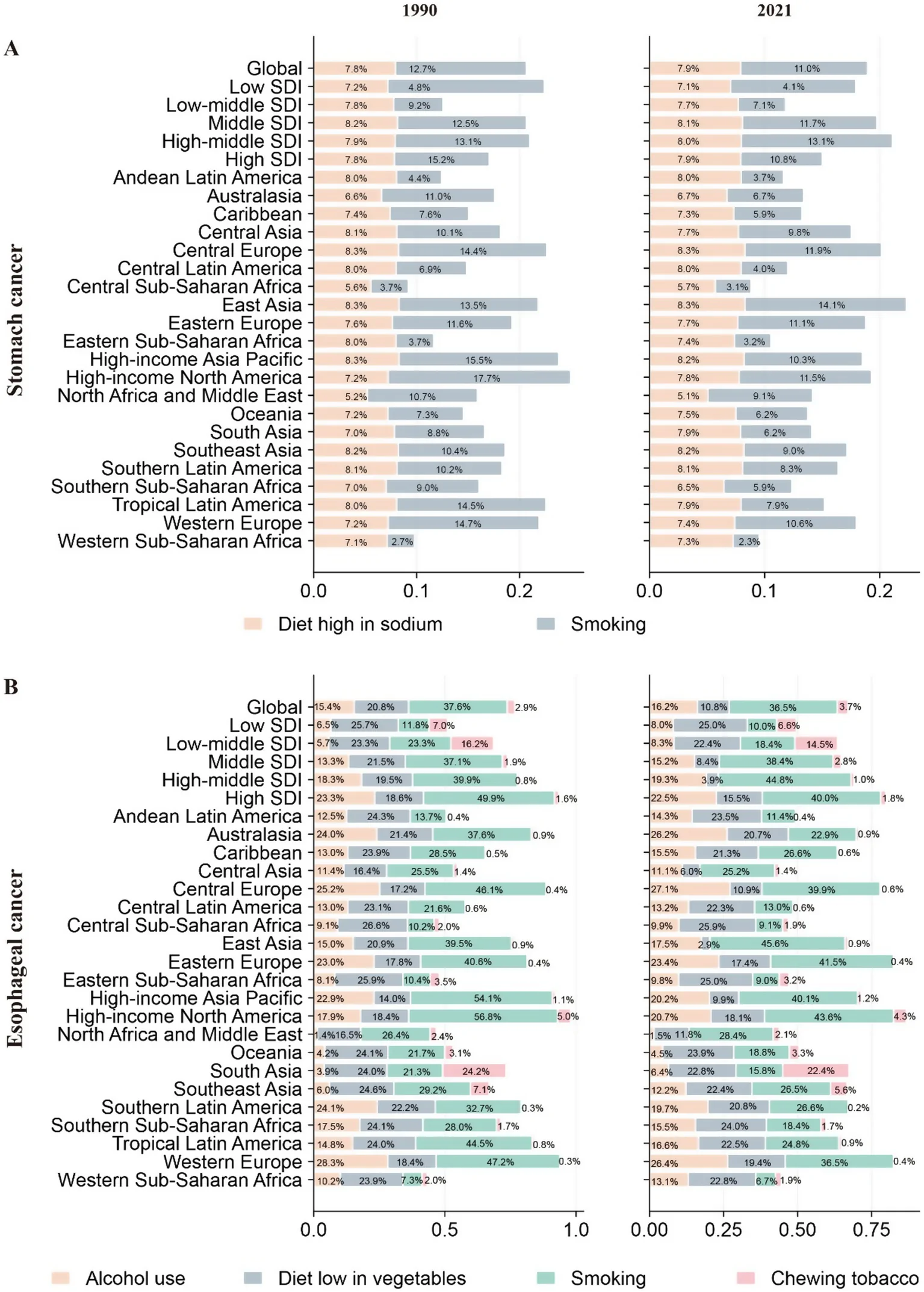
PAF statistics for risk factors of stomach and esophageal cancers in 1990 and 2021 at the global level, across five SDI levels, and 21 GBD regions. **(A)** Stomach cancer; **(B)** esophageal cancer. PAF, age-standardized Population Attributable Fraction; SDI, Socio-demographic Index.

**Figure 7:**
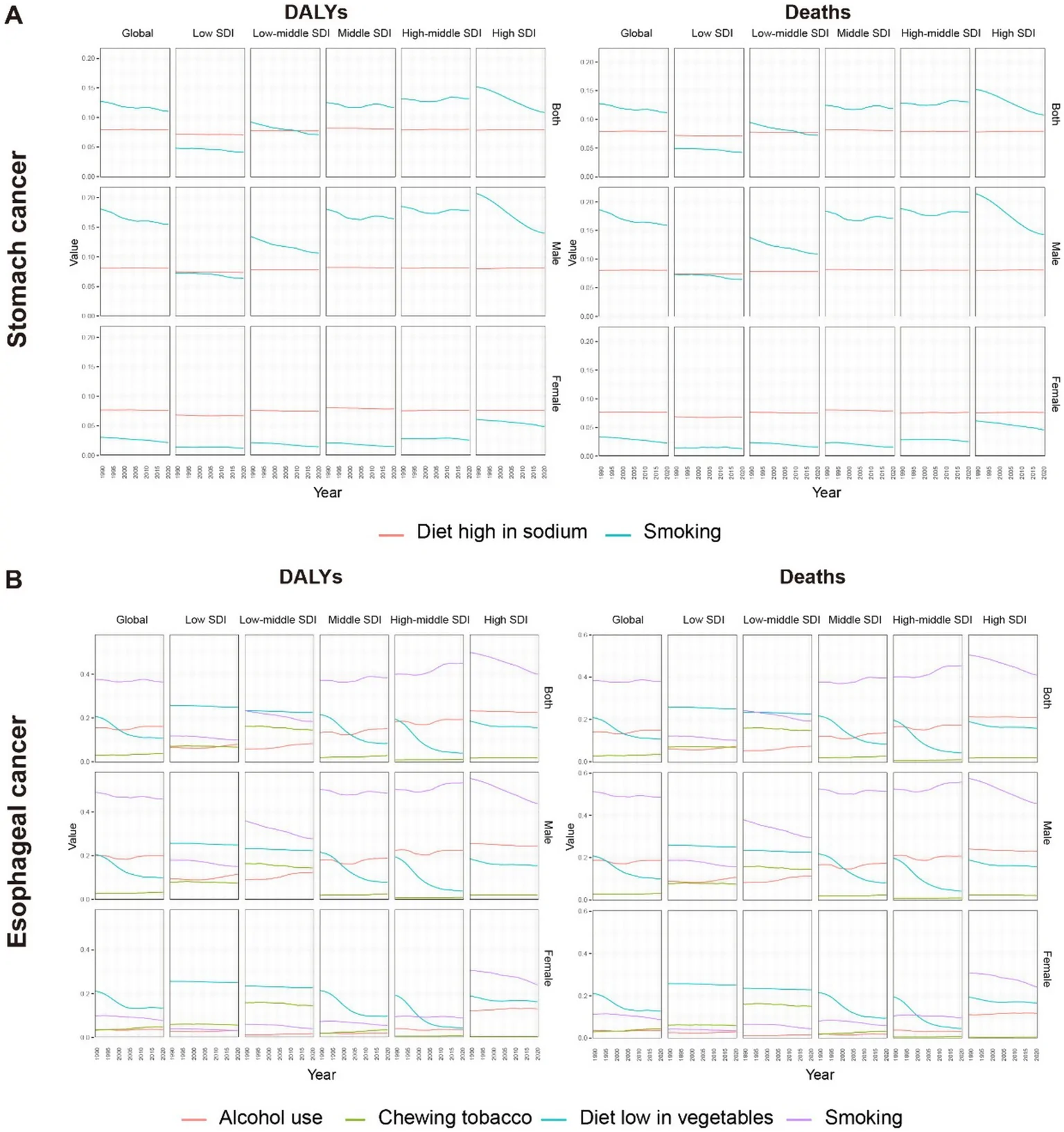
Yearly trends in PAFs for risk factors of stomach and esophageal cancers from 1990 to 2021 at the global level and across five SDI regions. **(A)** Stomach cancer; **(B)** esophageal cancer. PAF, age-standardized Population Attributable Fraction; SDI, Socio-demographic Index; DALYs, Disability-Adjusted Life Years.

Smoking PAFs were moderately to strongly positively correlated with SDI for both stomach and esophageal cancer, especially among males (e.g., *r* = 0.74 for esophageal cancer DALY in 1990). Alcohol use also showed a positive SDI correlation, while low vegetable intake and chewing tobacco were weakly negatively correlated with SDI. The latter showed a stronger correlation in females than in males (Supplementary Figures S10A,B; Supplementary Table S8).

## 4 Discussion

This study utilized the GBD 2021 dataset to provide a comprehensive analysis of the global burden and temporal trends of upper GI diseases—including GERD, PUD, GD, stomach cancer, and esophageal cancer—across 204 countries from 1990 to 2021. Through stratified analysis by age, sex, SDI, and geography, we identified disparities in disease burden, characterized major contributors to temporal changes, and projected future trends, offering evidence to inform targeted public health interventions.

Overall, disease burden varied markedly by region, sex, and age. While GERD burden increased globally, the burdens of PUD, GD, stomach cancer, and esophageal cancer generally declined. However, absolute burdens remained disproportionately higher in low SDI regions. Notably, the inequality in GERD and PUD burden has slightly decreased, and the burden of stomach cancer has shifted from high- to low SDI regions. In contrast, GD and esophageal cancer displayed worsening inequalities, particularly for ASIR and male ASPR in GD and ASDALY, ASMR, and ASIR in esophageal cancer. Sex-specific patterns revealed that males bore greater burdens of PUD, stomach cancer, and esophageal cancer, whereas females exhibited higher burdens of GERD and GD but showed better outcomes, particularly for GD. Older populations consistently experienced higher disease burdens, and peak age groups shifted toward older cohorts across all conditions.

Our decomposition analyses showed that demographic forces, particularly population growth and aging, were major contributors to changes in absolute disease burden, especially for GERD and esophageal cancer. In contrast, reductions in the absolute burden of PUD, GD, and stomach cancer were primarily driven by epidemiological improvements. These changes may partly reflect improvements in disease prevention and management, including Hp eradication, increased use of PPI, and expanded screening programs (26–28). Globally, GBD risk-attribution estimates identified several important modifiable risk factors. Smoking was the most prominent risk factor for stomach and esophageal cancers and showed strong positive correlations with SDI. The decline in smoking-attributable burden observed in high SDI regions may partly reflect tobacco-control efforts reported in these settings. Alcohol use emerged as the second leading risk factor for esophageal cancer, particularly in men and high SDI countries. Meanwhile, dietary risks, such as insufficient vegetable intake and high sodium diets, were especially burdensome in low SDI settings, where limited access to fresh produce persists (29–31). Chewing tobacco use, highly prevalent in South Asia, remains a significant risk factor, especially among women and lower SDI populations (32). In recent years, despite improvements, disparities still persist. Smoking-related PAFs for stomach and esophageal cancers declined in high SDI regions but rose in high-middle SDI countries, like rural China, where smoking remains prevalent. Alcohol-related burdens have also increased, particularly among middle-aged men in high SDI settings (33, 34). Chewing tobacco continues to be a culturally normalized practice in South Asia, exacerbated by misconceptions about its health benefits (32).

The rapid increase in GERD burden between 2015 and 2019, particularly in middle SDI regions, may be associated with rapid urbanization, lifestyle changes, obesity, and other behavioral factors reported in previous studies (35, 36). The relatively high GERD burden observed in parts of Latin America, including Brazil and Paraguay, may be associated with regional patterns of obesity and diet reported in previous studies (37, 38). In contrast, nations like Switzerland, with healthier diets and lower obesity rates, maintain a lower GERD burden (39, 40). Similarly, The high PUD burden observed in some countries, such as Cambodia and Kiribati, may be associated with high-salt and spicy diets (29, 31). The correlation between unhealthy behaviors and higher GI cancer burden among males may reflect broader trends in smoking and alcohol consumption (41, 42).

Differences in healthcare infrastructure may contribute to the eographic disparities observed in upper GI disease burden. In low SDI regions, limited healthcare resources may be associated with later diagnoses and poorer outcomes, whereas greater access to Hp testing and treatment, PPIs, and organized cancer screening in higher SDI settings may partly contribute to the observed differences (43–45). Notably, PUD and GD burden began declining rapidly after 2004, coincided with the broader adoption of Hp eradication and standardized pharmacological management reported during this period (26, 27). The decline in esophageal cancer burden since 2017 may partly coincide with improvements in early detection and screening reported in high-risk regions (46, 47).

Sex hormones and physiological differences may partly contribute to the observed sex-specific patterns. Estrogen offers mucosal protection against ulcers and cancer but lowers lower esophageal sphincter tone, which may partly contribute to sex differences in susceptibility to GERD (48, 49). Males are more likely to experience severe disease progression and higher mortality. Aging significantly contributes to all upper GI diseases, with immune senescence, impaired mucosal repair, and cumulative carcinogen exposure elevating the burden, especially for stomach and esophageal cancers in older adults (50, 51). The decline after the peak at ages 85–89 may partly reflect survivor effects or greater uncertainty in estimates at very advanced ages.

Demographic changes have influenced disease burden trends. From 1990 to 2021, the global population grew by over 2.5 billion, increasing the absolute burden of GI diseases, especially GERD and esophageal cancer in low and low-middle SDI regions (52). Population aging contributed more substantially to increases in absolute disease burden in middle- and high-middle SDI regions. In Sub-Saharan Africa, high fertility rates continue to drive population growth, increasing the absolute disease burden and place additional pressure on healthcare systems (53). Compared to earlier global reports, several new trends have emerged. The burden of GERD and PUD has resurged in low SDI regions despite treatment and diagnostic improvements, and the peak burden age has shifted older. For instance, the ASDALY peak for GD shifted from 85 to 89 years in 2019 to 95 + years in 2021, which may be related to survival and chronic disease care access. Meanwhile, age-specific incidence rates of esophageal cancer has increased in younger age groups, which may reflect changing exposure patterns or shifts in early-onset disease (9, 54).

Projection analyses indicate that GERD burden will continue to increase, with males expected to experience a more rapid increase despite the higher overall burden in females. Together with the observed increase in middle SDI regions and the higher burden among women and older adults, these findings support prioritizing weight management, dietary modification, and other reflux-related lifestyle interventions in middle SDI regions, particularly among women and older adults. For PUD, although ASMR and ASDALY are projected to decline, ASIR and ASPR may increase slightly, while males are expected to maintain a higher burden. Continued access to Hp eradication and PPI therapy should therefore be prioritized, together with strengthened prevention and management of ulcer-related complications, particularly among older men and in lower SDI regions. For GD, the projected decline across all indicators should not obscure the persistent burden among older adults and marked sex-specific disparities. Prevention and management efforts should remain focused on middle-aged and older adults, with greater emphasis on early detection and standardized disease management in women, who showed a higher prevalence, and on timely diagnosis and treatment in men to reduce their higher mortality burden. For stomach and esophageal cancers, although ASRs are projected to decline, males are expected to maintain a higher disease burden. For stomach cancer, regional prevention efforts should prioritize low SDI settings, with particular emphasis on smoking cessation in men and population-level sodium reduction given the persistent contribution of high-sodium diets (55, 56). For esophageal cancer, smoking cessation should remain a major priority, while alcohol reduction should be strengthened particularly among middle-aged adults in high SDI settings, where alcohol-attributable burden increased. In South Asia, chewing-tobacco cessation and control should be further strengthened, especially among adults aged 30–44 years (32, 57). Increasing vegetable intake should also be encouraged in populations where low vegetable consumption remains an important attributable risk (58).

This study offers a comprehensive, global overview of upper GI disease burden over decades and populations, providing actionable insights for health systems and governments. Nevertheless, several limitations should be acknowledged. First, the availability and quality of primary data remain limited in some low-income settings, where GBD estimates rely more heavily on statistical modeling and extrapolation. Second, the GBD framework used in this study did not allow systematic urban–rural comparisons, which may obscure important within-country disparities. Third, the risk factor analysis was restricted to factors available in the GBD 2021 framework and therefore did not capture the full spectrum of relevant environmental, behavioral, infectious, and metabolic determinants of upper GI diseases. Finally, the long-term projections were based on historical trends and model assumptions and were not subjected to retrospective out-of-sample validation. Therefore, they should be interpreted as exploratory estimates rather than precise forecasts of future disease burden. They cannot fully account for future changes in disease determinants, healthcare systems, public health policies, or advances in diagnosis, prevention, and treatment.

## Data Availability

Publicly available datasets were analyzed in this study. This data can be found here: the datasets analysed during the current study are publicly available from the Global Burden of Disease (GBD) database at http://ghdx.healthdata.org/gbd-results-tool.
