# Supplementary material for: Global burden, trends, and projections of five major upper gastrointestinal diseases, 1990–2046: an analysis of GBD 2021
Source: Front Public Health. 2026 Oct 15;14:1898446. doi: 10.3389/fpubh.2026.1898446 (PMC13716786; doi:10.3389/fpubh.2026.1898446)
Supplement: Supplementary file 1 [file Supplementary_file_1.docx]

Supplementary Figures

**Figure S1.** Concentration indices of regional inequality in five upper gastrointestinal diseases in 1990 and 2021.

DALYs: Disability-Adjusted Life Years; The colors indicate the reference years: green for 1990 and purple for 2021. Point estimates are shown in the figure, and the corresponding 95% confidence intervals are provided in Table S3.

**Figure S2.** Slope indices of regional inequality in five upper gastrointestinal diseases in 1990 and 2021.

ASRs: age-standardized rates; DALYs: Disability-Adjusted Life Years; The colors indicate the reference years: green for 1990 and purple for 2021. Point estimates are shown in the figure, and the corresponding 95% confidence intervals are provided in Table S4.

**Figure S3.** Age-specific distribution of case numbers and rates for five upper gastrointestinal diseases in 1990 and 2021, stratified by sex.

DALYs: Disability-Adjusted Life Years; The line graph represents age-specific rates, and the bar chart represents absolute numbers. Red indicates females, and blue indicates males.

**Figure S4.** Temporal trends in ASRs of five upper gastrointestinal diseases from 1990 to 2021, stratified by sex.

ASRs: age-standardized rates; DALYs: Disability-Adjusted Life Years.

**Figure S5.** Joinpoint analysis of ASRs for five upper gastrointestinal diseases from 1990 to 2021.

ASRs: age-standardized rates; DALYs: Disability-Adjusted Life Years; APC: annual percent change; AAPC: average annual percent change.

**Figure S6.** Distribution of the EAPC for five upper gastrointestinal diseases across 204 countries and regions from 1990 to 2021.

EAPC: estimated average annual percent change; EAPCs are presented as point estimates; corresponding 95% confidence intervals are provided in the Supplementary Tables.

**Figure S7.** Projected ASRs of five upper gastrointestinal diseases from 2022 to 2046.

DALYs: Disability-Adjusted Life Years; ASRs: age-standardized rates; Solid lines represent observed estimates and dashed lines represent projected estimates. Corresponding prediction intervals are provided in Table S7.

**Figure S8.** PAF statistics for risk factors of stomach and esophageal cancers in 1990 and 2021 at the global level, across five SDI levels and 21 GBD regions.

PAF: age-standardized Population Attributable Fraction; SDI: Socio-demographic Index; DALYs: Disability-Adjusted Life Years.

**Figure S9.** Yearly trends in PAFs for risk factors of stomach and esophageal cancers from 1990 to 2021 across different age groups.

PAF: age-standardized Population Attributable Fraction; SDI: Socio-demographic Index; DALYs: Disability-Adjusted Life Years.

**Figure S10.** Regression analysis of PAFs and SDI for risk factors of stomach and esophageal cancers in 1990 and 2021.

PAF: age-standardized Population Attributable Fraction; DALYs: Disability-Adjusted Life Years; SDI: Socio-demographic Index.

**Figure S1.** Concentration indices of regional inequality in five upper gastrointestinal diseases in 1990 and 2021.

DALYs: Disability-Adjusted Life Years; The colors indicate the reference years: green for 1990 and purple for 2021. Point estimates are shown in the figure, and the corresponding 95% confidence intervals are provided in Table S3.

**Figure S2.** Slope indices of regional inequality in five upper gastrointestinal diseases in 1990 and 2021.

ASRs: age-standardized rates; DALYs: Disability-Adjusted Life Years; The colors indicate the reference years: green for 1990 and purple for 2021. Point estimates are shown in the figure, and the corresponding 95% confidence intervals are provided in Table S4.

**Figure S3.** Age-specific distribution of case numbers and rates for five upper gastrointestinal diseases in 1990 and 2021, stratified by sex.

DALYs: Disability-Adjusted Life Years; The line graph represents age-specific rates, and the bar chart represents absolute numbers. Red indicates females, and blue indicates males.

**Figure S4.** Temporal trends in ASRs of five upper gastrointestinal diseases from 1990 to 2021, stratified by sex.

ASRs: age-standardized rates; DALYs: Disability-Adjusted Life Years.

**Figure S5.** Joinpoint analysis of ASRs for five upper gastrointestinal diseases from 1990 to 2021.

ASRs: age-standardized rates; DALYs: Disability-Adjusted Life Years; APC: annual percent change; AAPC: average annual percent change.

**Figure S6.** Distribution of the EAPC for five upper gastrointestinal diseases across 204 countries and regions from 1990 to 2021.

EAPC: estimated average annual percent change; EAPCs are presented as point estimates; corresponding 95% confidence intervals are provided in the Supplementary Tables.

**Figure S7.** Projected ASRs of five upper gastrointestinal diseases from 2022 to 2046.

DALYs: Disability-Adjusted Life Years; ASRs: age-standardized rates; Solid lines represent observed estimates and dashed lines represent projected estimates. Corresponding prediction intervals are provided in Table S7.

**Figure S8.** PAF statistics for risk factors of stomach and esophageal cancers in 1990 and 2021 at the global level, across five SDI levels and 21 GBD regions.

PAF: age-standardized Population Attributable Fraction; SDI: Socio-demographic Index; DALYs: Disability-Adjusted Life Years.

**Figure S9.** Yearly trends in PAFs for risk factors of stomach and esophageal cancers from 1990 to 2021 across different age groups.

PAF: age-standardized Population Attributable Fraction; SDI: Socio-demographic Index; DALYs: Disability-Adjusted Life Years.

**Figure S10.** Regression analysis of PAFs and SDI for risk factors of stomach and esophageal cancers in 1990 and 2021.

PAF: age-standardized Population Attributable Fraction; DALYs: Disability-Adjusted Life Years; SDI: Socio-demographic Index.
